# Impact of Prior Ipsilateral Arthrodesis on Subsequent Ankle and Subtalar Fusion Outcomes: A Propensity-Matched Cohort Study

**DOI:** 10.1177/10711007251376296

**Published:** 2025-11-05

**Authors:** Avani A. Chopra, Kush Mody, Mark Fisher, David Ahn, Gnaneswar Chundi, Abhiram Dawar, Tuckerman Jones, Scott Tucker, Sheldon Lin, Michael Aynardi

**Affiliations:** 1 Penn State College of Medicine, Hershey, PA, USA; 2 Rutgers New Jersey Medical School, Newark, NJ, USA

**Keywords:** ankle arthrodesis, subtalar arthrodesis, nonunion

## Abstract

**Background:**

Although prior ankle or subtalar arthrodesis is thought to affect outcomes at adjacent joints, previous studies have not distinguished between successful and failed prior fusions. This study examines whether prior successful vs failed ipsilateral arthrodesis influences nonunion risk in subsequent ankle or subtalar fusion. The primary objective of this study is to examine nonunion rates after subtalar and ankle arthrodesis in patients with and without prior ipsilateral arthrodesis, and vice versa.

**Methods:**

A retrospective study using the TriNetX Research Network compared nonunion rates in patients who underwent subtalar (ST) arthrodesis with (ankle-ST) or without (ST-only) prior ankle arthrodesis. Patients were stratified by whether the prior arthrodesis was successful (no nonunion diagnosis/revision) or failed (nonunion requiring revision Ankle-ST patients were stratified by successful or failed prior ankle arthrodesis. A secondary analysis evaluated ankle arthrodesis in patients with (ST-ankle) or without (ankle-only) prior subtalar arthrodesis, similarly stratified. Propensity score matching (1:1) adjusted for age, sex, body mass index (BMI), and comorbidities. The primary outcome is nonunion rates.

**Results:**

144 patients were in the successful ankle-ST cohort and 12,635 in the ST-only cohort. After propensity score matching, successful ankle-ST patients had no difference in nonunion rates (16.7% vs 16.0%, *P* = .873). However, patients with failed prior ankle arthrodesis had a 3-fold higher risk of subtalar nonunion compared to those with successful ankle fusion (risk ratio [RR] 3.0, 95% CI 1.79-5.02). Furthermore, 109 patients were in the successful ST-Ankle group and 6,801 in the ankle-only group. After matching, there was no difference in rate of nonunion (11.1% vs 19.4%, *P* = .089) between the successful ST-ankle and the ankle-only groups. Conversely, patients with failed prior subtalar arthrodesis had a 2.4-fold higher risk of ankle nonunion compared to those with successful subtalar fusion (RR 2.4, 95% CI 1.30-4.42).

**Conclusion:**

Our analysis of the TriNetX Research Network database suggests that when the primary ankle or subtalar arthrodesis is successful, performing a subsequent adjacent fusion does not significantly increase the risk of nonunion compared with an isolated fusion. However, failed prior arthrodesis substantially increases nonunion risk, highlighting the importance of distinguishing between successful and failed prior procedures in clinical decision-making.

## Graphical abstract

**Graphical Abstract:**
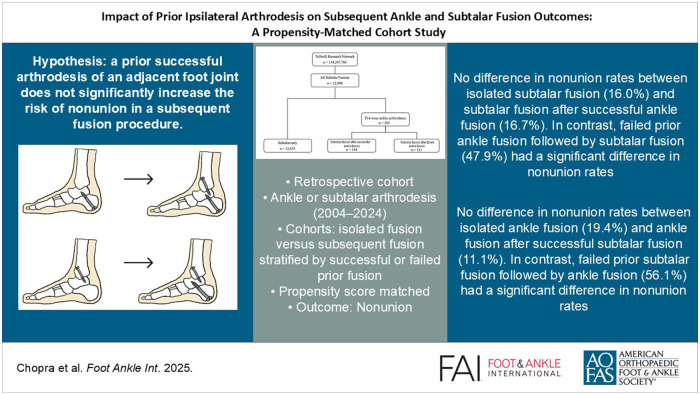
This is a visual representation of the abstract.

**Level of Evidence:** Level III, restrospective cohort study.

## Introduction

Subtalar arthrodesis is a widely performed procedure for managing arthritis, calcaneal fractures, deformities, and instability of the hindfoot.^7^ Reported fusion rates for subtalar arthrodesis range from 60% to 96%, with successful outcomes attributed to advancements in fixation techniques, bone grafting, and postoperative rehabilitation protocols.^2,7,9,11-14,22,23^ Some studies suggest that prior ankle arthrodesis may negatively affect subtalar fusion due to altered joint biomechanics and vascular supply, but evidence remains limited.^5,8,19,23^ Although both ankle and subtalar arthrodesis are commonly performed procedures for end-stage arthritis and deformity, limited evidence exists on how prior fusion at one joint affects outcomes at the adjacent joint. In particular, the risk of nonunion in secondary ipsilateral arthrodesis remains poorly defined. Previous studies addressing this question have been limited by small sample sizes and underpowered analyses, often including fewer than 20 patients with prior procedures, making it difficult to draw definitive conclusions. Furthermore, limited data exist regarding outcomes of ankle arthrodesis following prior subtalar fusion, as few studies isolate this patient population.

Our assumption is that a successful prior fusion should not impact healing at an adjacent joint. One proposed counterargument is that prior surgery may disrupt local vascular supply, potentially affecting osseous healing. However, from an anatomical standpoint, the ankle and subtalar joints are separated by approximately 3-4 cm, and the regional blood supply to each joint is largely independent.

The primary goal of the study was to compare nonunion rates between patients undergoing subtalar or ankle arthrodesis with and without a prior ipsilateral arthrodesis. We hypothesize that a prior successful arthrodesis of an adjacent foot joint does not significantly increase the risk of nonunion in a subsequent fusion procedure. We propose that it is the failure of the prior fusion that contributes to increased risk of nonunion following subsequent ipsilateral fusion, an important distinction not made in prior studies. This study evaluates that hypothesis using a large, matched cohort with adequate statistical power to address this clinically relevant question. Our findings help clarify the impact of prior arthrodesis on subsequent adjacent joint fusion success and guide surgical decision making.

## Methods

Data for this study were obtained from the TriNetX Research Network, a global federated health research platform that aggregates deidentified electronic medical records from health care organizations, including academic medical centers, specialty physician networks, and community hospitals. The network includes patient-level longitudinal data such as demographics, diagnoses, procedures, medications, and laboratory values. All data are deidentified in compliance with the Health Insurance Portability and Accountability Act (HIPAA) Privacy Rule.

This retrospective cohort study used data collected on February 22, 2025, from the TriNetX Research Network, which provided access to electronic medical records from approximately 140 million patients. This study is exempt from Institutional Review Board approval. The data reviewed is a secondary analysis of existing data, does not involve intervention or interaction with human subjects, and is deidentified.

**Figure 1.**
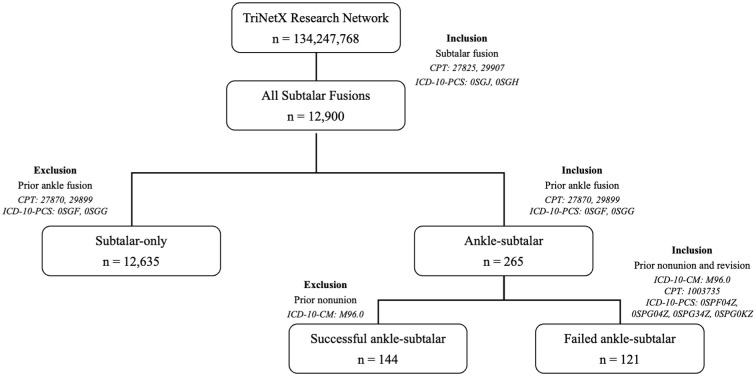
Flowchart depicting cohort design for Ankle-ST and ST-only cohorts.

The TriNetX Research Database was queried to identify patients who underwent subtalar arthrodesis between February 20, 2004, and February 20, 2024, using *Current Procedural Terminology* (*CPT*) codes 29907 and 28725 and *International Classification of Diseases, Tenth Revision* (*ICD-10*) codes 0SGJ and 0SGH. Only patients with a minimum of 1 year of follow-up were included. Based on surgical history and timing, patients were categorized into 2 cohorts (Figure 1).

For the subtalar arthrodesis analysis, patients were categorized as “ST-only” (those who underwent isolated subtalar arthrodesis) or “Ankle-ST” (those who later underwent subtalar arthrodesis following a prior ankle fusion). For the ankle arthrodesis analysis, patients were classified as “Ankle-only” (isolated ankle arthrodesis) or “ST-Ankle” (those who later underwent ankle arthrodesis following subtalar fusion).

To ensure accurate sequencing of procedures, the order of operations was determined using procedure dates. Patients were included only if the second arthrodesis occurred after the index procedure and met all temporal and clinical inclusion criteria.

The ST-only cohort included patients with no prior history of ipsilateral ankle arthrodesis. The Ankle-ST cohort consisted of patients who had undergone ipsilateral ankle arthrodesis between 6 months and 5 years before their subtalar arthrodesis; this group included both patients with a successful prior ankle fusion (successful ankle-ST) and those who experienced a failed ankle fusion requiring a healed revision prior to the subtalar arthrodesis (failed ankle-ST).

A secondary analysis was conducted within the ankle-ST arthrodesis vs ST cohorts, comparing outcomes between patients with successful Ankle-ST vs ST-only, and between those with failed Ankle-ST vs ST-only.

A prior ankle arthrodesis was considered successful if no nonunion diagnosis (*ICD-10*: M96.0) as well as no revision procedure was recorded within 5 years preceding the subsequent subtalar arthrodesis. A failed ankle arthrodesis was defined by the presence of both a nonunion diagnosis and a subsequent successful revision procedure within 5 years preceding the subtalar arthrodesis. To reduce heterogeneity in healing response and biomechanical adaptation, we limited inclusion to patients who underwent the secondary arthrodesis within 6 months to 5 years of the index procedure. Additionally, the 5-year window was selected as it represented the minimal possible interval between procedures to achieve cohorts of substantial enough size.

**Figure 2.**
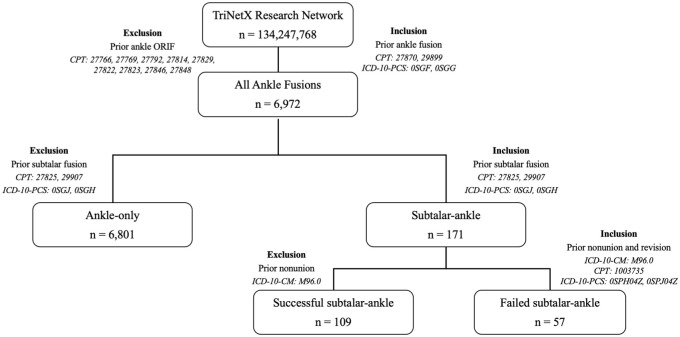
Flowchart depicting cohort design for the ST-Ankle and Ankle-only cohorts.

A second query was run to identify patients who underwent ankle arthrodesis between February 20, 2004, and February 20, 2024, using *CPT* codes 27870 and 29899 and *ICD-10* codes 0SGF and 0SGG. Only patients with a minimum of 1 year of follow-up were included. Based on surgical history and timing, patients were categorized into 2 different cohorts (Figure 2). The Ankle-only cohort included patients who had ankle arthrodesis without any history of ipsilateral subtalar arthrodesis. The ST-Ankle cohort comprised patients who had an ipsilateral subtalar arthrodesis between 6 months and 5 years before their ankle arthrodesis; this group included both patients with a successful prior subtalar fusion (successful ST-ankle) and those who experienced a subtalar nonunion requiring a healed revision prior to the ankle arthrodesis (failed ST-ankle).

A secondary analysis was conducted within the ST-ankle arthrodesis vs ankle cohorts, comparing outcomes between patients with successful ST-ankle vs Ankle-only cohorts, and between those with failed ST-ankle vs Ankle-only.

A prior subtalar arthrodesis was considered successful if no nonunion diagnosis (*ICD-10*: M96.0) was recorded within 5 years preceding the subsequent ankle arthrodesis. This code was selected because of its specificity in capturing pseudarthrosis as a postoperative complication. Although *ICD* codes are proxies and not substitutes for radiographic or clinical confirmation, prior studies have used this code to estimate postoperative nonunion in large data sets.^4,6^ A failed prior subtalar fusion was defined by the presence of both a nonunion diagnosis and a subsequent revision procedure (*CPT*: 20670, 20680; *ICD-10*: 0SPH04Z, 0SPJ04Z) within 5 years preceding the ankle arthrodesis.

A minimum temporal window of 6 months between index and subsequent procedure was chosen, because 6 months represents the minimum time necessary to diagnose nonunion. A maximum temporal window of 5 years was used to ensure adequate sample size according to our post hoc power analyses that confirmed ≥80% power to detect differences in nonunion rates at this sample size.^16^

Propensity score matching was performed to reduce the impact of confounding variables and ensure balanced comparisons between cohorts. Patients were matched in a 1:1 ratio using greedy nearest-neighbor matching with a caliper of 0.1 pooled standard deviations, and propensity scores were estimated using logistic regression. The covariates included in the matching process were age, sex, tobacco use history, and select comorbidities. Comorbidities included are shown in Table 1. Matching was conducted with a tolerance of 0.01 to minimize discrepancies in propensity scores between matched pairs. All covariates, including current smoking status, had postmatch standardized mean difference (SMD) < 0.20, with slight deviations above 0.10 attributable to TriNetX’s fixed 0.1 caliper matching algorithm and, for select variables, data suppression rules for cell counts <10 rather than poor balance.

**Table 1.** Comparison of Patient Demographics and Comorbidities Between Ankle-Subtalar and Subtalar-Only Cohorts Before and After Propensity Score Matching.

Characteristics	Before Matching				After Matching			
	Ankle-Subtalar	Subtalar-Only	*P* Value	SMD	Ankle-Subtalar	Subtalar-Only	*P* Value	SMD
Age, y, mean	54.8	52.3	.013	0.17	54.8	55.0	.854	0.02
BMI, mean	34.1	32.5	.002	0.23	34.1	33.0	.106	0.16
Male, n (%)	138 (51.5)	4492 (43.3)	.008	0.16	137 (51.3)	144 (53.9)	.544	0.05
Female, n (%)	120 (44.8)	5363 (51.7)	.025	0.14	120 (44.9)	118 (44.2)	.862	0.02
Acute myocardial infarction, n (%)	<10 (3.7)^a^	60 (0.6)	<.001	0.22	<10 (3.7)^a^	0 (0.0)	.001	0.28
Cancer, n (%)	21 (7.8)	942 (9.1)	.483	0.04	21 (7.9)	21 (7.9)	>.999	0.00
Cerebral vascular accident, n (%)	<10 (3.7)^a^	85 (0.8)	<.001	0.20	<10 (3.7)^a^	0 (0.0)	.001	0.28
Congestive heart failure, n (%)	17 (6.3)	239 (2.3)	<.001	0.20	16 (6.0)	12 (4.5)	.437	0.07
Connective tissue disorder, n (%)	<10 (3.7)^a^	190 (1.8)	.024	0.12	<10 (3.7)^a^	<10 (3.7)^a^	>.999	0.00
Dementia, n (%)	0 (0.0)	11 (0.1)	.594	0.05	0 (0.0)	0 (0.0)	-	-
Diabetes mellitus, n (%)	68 (25.4)	1582 (15.2)	<.001	0.25	67 (25.1)	53 (19.9)	.147	0.13
Hemiplegia, n (%)	0 (0.0)	37 (0.4)	.327	0.08	0 (0.0)	0 (0.0)	-	-
HIV, n (%)	<10 (3.7)^a^	31 (0.3)	<.001	0.25	<10 (3.7)^a^	<10 (3.7)^a^	>.999	0.00
Liver disease, n (%)	11 (4.1)	285 (2.7)	.182	0.07	11 (4.1)	<10 (3.7)^a^	.824	0.02
Peptic ulcer, n (%)	<10 (3.7)^a^	44 (0.4)	<.001	0.23	<10 (3.7)^a^	<10 (3.7)^a^	>.999	0.00
Peripheral vascular disease, n (%)	14 (5.2)	330 (3.2)	.062	0.10	14 (5.2)	15 (5.6)	.849	0.02
Pulmonary disease, n (%)	44 (16.4)	1052 (10.1)	<.001	0.19	43 (16.1)	46 (17.2)	.728	0.03
Renal disease, n (%)	27 (10.1)	432 (4.2)	<.001	0.23	26 (9.7)	22 (8.2)	.545	0.05
Smoking status, n (%)	38 (14.2)	840 (8.1)	<.001	0.19	37 (13.9)	48 (18.0)	.193	0.11
Estimated CCI	350	6,886	-	-	345	302	-	-

Abbreviations: CCI, Charlson Comorbidity Index; HIV, Human immunodeficiency virus; SMD, standardized mean difference.

a TriNetX does not provide exact numbers if less than 10 to protect against identification.

We collected data on patient demographics, tobacco use, and comorbidities. The Charlson Comorbidity Index (CCI) was calculated as a measure of total comorbidity burden for each cohort. CCI scores were calculated according to standard methodology; however, because of limitations of the TriNetX database, the weights assigned to each condition were not adjusted for disease severity (Supplemental 1). The primary outcome of this study was the 1-year rate of nonunion following subsequent arthrodesis, which was assessed using matched cohorts. Nonunion was defined using the *ICD-10* diagnosis code M96.0. For health systems providing *ICD-9-CM* data, TriNetX applies General Equivalence Mappings (GEMs) and custom curation to convert to *ICD-10-CM*. As TriNetX uses structured electronic medical record data, this diagnosis must be clinician-entered, likely based on appropriate imaging and clinical evaluation. Although some misclassification is possible, we believe it is rare given standard diagnostic practices.

Demographic characteristics and postoperative outcomes were compared across cohorts using χ^2^ tests for categorical variables and *t* tests for continuous variables. The risk of experiencing outcomes was evaluated by calculating risk differences with 95% CIs, and *Z* tests were used to determine the significance of these risk differences. Significance threshold was set at *P* <.05. Risk ratios (RRs) were calculated to assess the impact of prior nonunion on future arthrodesis outcomes. All statistical analysis was performed using the TriNetX platform, which uses Java, R, and Python for statistical computing.

To minimize misclassification bias, patients who underwent triple or double arthrodesis procedures were excluded to simplify the cohort and reduce potential confounding from multijoint procedures. Tibiotalocalcaneal (TTC) fusion cases—identified by concurrent coding of ankle and subtalar fusion on the same day—were excluded to avoid misclassification bias and ensure the separation of ankle and subtalar fusion cohorts. For the ST-only group, we excluded any patients with a history of prior ankle fusion, using both *CPT* and *ICD-10* code histories to ensure cohort purity. Additionally, to reduce confounders from remote ankle fusions (performed more than 5 years before the index date), we refined our inclusion criteria to include only those with temporally relevant prior procedures. These criteria are detailed to improve specificity and reduce potential sources of bias in the analysis.

## Results

### Ankle-ST vs ST-Only

A total of 265 patients in the Ankle-ST cohort and 12 635 patients in the ST-only cohort were identified before matching. The following is a comparison of characteristics of the ST cohort vs the Ankle-ST cohort before matching. The Ankle-ST cohort had a significantly higher mean BMI (34.1 vs 32.5, *P* = .002) and higher prevalence of diabetes mellitus (25.4% vs 15.2%, *P* < .001), pulmonary disease (16.4% vs 10.1%, *P* < .001), renal disease (10.1% vs 4.2%, *P* < .001), heart failure (6.3% vs 2.3%, *P* < .001), and tobacco use (14.2% vs 8.1%, *P* < .001). After propensity score matching, each group consisted of 262 patients (Table 1). The Ankle-ST cohort had significantly higher rates of subtalar nonunion (30.5% vs 18.7%, *P* = .002) than the ST-only cohort, therefore rejecting the null hypothesis. The Ankle-ST group had a 1.6 times greater risk of developing nonunion after subsequent ST arthrodesis than the ST-only group (RR = 1.6, 95% CI = 1.196-2.230) (Table 3).

Of the 265 patients in the Ankle-ST group, 144 patients had a successful primary ankle fusion, 121 had a failed primary ankle arthrodesis requiring revision, and none had a failed primary ankle arthrodesis without revision.

In the subanalysis of the successful ankle-ST cohort, the 144 patients were compared to patients in the ST-only cohort. Before matching, the successful ankle-ST cohort had a significantly higher mean BMI compared with the ST-only cohort (34.7 vs 32.3, *P* = .001) and a higher prevalence of diabetes mellitus (27.1% vs 15.6%, *P* < .001), pulmonary disease (16.7% vs 10.4%, *P* = .014), renal disease (11.1% vs 4.5%, *P* < .001), and tobacco use (15.3% vs 8.2%, *P* = .002). After propensity score matching, each group consisted of 144 patients (Supplemental 2). There was no difference in rates of nonunion (16.7% vs 16.0%, *P* = .873) between the successful ankle-ST and the ST-only groups, therefore failing to reject the null hypothesis.

Next, a total of 121 patients in the failed ankle-ST group were identified and compared to the ST-only cohort. Before matching, the Ankle-ST cohort had a significantly higher mean BMI (34.1 vs 32.3, *P* = .023) and a higher prevalence of diabetes mellitus (23.8% vs 15.6%, *P* = .014), pulmonary disease (22.1% vs 10.4%, *P* < .001), and renal disease (10.7% vs 4.5%, *P* = .001). After propensity score matching, each group consisted of 121 patients (Supplemental 3). The failed ankle-ST arthrodesis cohort had a significantly higher rate of nonunion (47.9% vs 14.9%, *P* < .001).

Additionally, the failed ankle-ST group had a 3 times greater risk of developing nonunion after subsequent subtalar arthrodesis than the successful Ankle-ST group (RR = 3.0, 95% CI = 1.79-5.02).

### ST-Ankle vs Ankle-only

A total of 171 patients in the ST-Ankle cohort and 6801 patients in the Ankle-only cohort were identified before matching. The ST-Ankle cohort had a higher prevalence of diabetes mellitus (24.2% vs 14.1%, *P* < .001), pulmonary disease (16.5% vs 8.9%, *P* < .001), and cancer (5.2% vs 1.8%, *P* < .001). After propensity score matching, each group consisted of 171 patients (Table 2). The ST-Ankle cohort had a notably higher rate of nonunion (27.5% vs 19.3%, *P* = .074) than the Ankle-only cohort, although it was not statistically significant. The ST-Ankle group had a 1.4 times greater risk of developing nonunion after subsequent ankle arthrodesis than the Ankle-only group (RR = 1.4, 95% CI = 0.963-2.107) (Table 4).

**Table 2.** Comparison of Patient Demographics and Comorbidities Between Subtalar-Ankle and Ankle-Only Cohorts Before and After Propensity Score Matching.

Characteristics	Before Matching				After Matching			
	Subtalar-Ankle	Ankle Only	*P* Value	SMD	Subtalar-Ankle	Ankle Only	*P* Value	SMD
Age (y), mean	54.5	54.7	.882	0.01	54.6	55.6	.476	0.07
BMI, mean	32.1	32.2	.882	0.02	31.8	32.9	.323	0.15
Male, n (%)	87 (44.8)	3385 (54.4)	.008	0.19	87 (45.5)	87 (45.5)	>.999	0.00
Female, n (%)	103 (53.1)	2681 (43.1)	.006	0.20	100 (52.4)	100 (52.4)	>.999	0.00
Acute myocardial infarction, n (%)	0 (0.0)	19 (0.3)	.441	0.08	0 (0.0)	0 (0.0)	-	
Cancer, n (%)	<10 (5.2)^a^	111 (1.8)	<.001	0.18	<10 (5.2)^a^	<10 (5.2)^a^	>.999	0.00
Cerebral vascular accident, n (%)	0 (0.0)	<10 (0.2)^a^	.576	0.06	0 (0.0)	0 (0.0)	-	
Congestive heart failure, n (%)	<10 (5.2)^a^	129 (2.1)	.004	0.17	<10 (5.2)^a^	<10 (5.2)^a^	>.999	0.00
Connective tissue disorder, n (%)	<10 (5.2)^a^	59 (0.9)	<.001	0.25	<10 (5.2)^a^	0 (0.0)	.001	0.33
Dementia, n (%)	0 (0.0)	<10 (0.2)^a^	.576	0.06	0 (0.0)	0 (0.0)	-	
Diabetes mellitus, n (%)	47 (24.2)	879 (14.1)	<.001	0.26	44 (23.0)	44 (23.0)	>.999	0.00
Hemiplegia, n (%)	0 (0.0)	<10 (0.2)^a^	.576	0.06	0 (0.0)	0 (0.0)	-	
HIV, n (%)	0 (0.0)	<10 (0.2)^a^	.576	0.06	0 (0.0)	0 (0.0)	-	
Liver disease, n (%)	<10 (5.2)^a^	60 (1.0)	<.001	0.25	<10 (5.2)^a^	<10 (5.2)^a^	>.999	0.00
Peptic ulcer, n (%)	<10 (5.2)^a^	<10 (0.2)^a^	<.001	0.31	<10 (5.2)^a^	0 (0.0)	.001	0.33
Peripheral vascular disease, n (%)	<10 (5.2)^a^	56 (0.9)	<.001	0.25	<10 (5.2)^a^	<10 (5.2)^a^	>.999	0.00
Pulmonary disease, n (%)	32 (16.5)	554 (8.9)	<.001	0.23	29 (15.2)	32 (16.8)	.675	0.04
Renal disease, n (%)	<10 (5.2)^a^	260 (4.2)	.505	0.05	<10 (5.2)^a^	<10 (5.2)^a^	>.999	0.00
Tobacco use, n (%)	14 (7.2)	491 (7.9)	.731	0.03	14 (7.3)	11 (5.8)	.535	0.06
Estimated CCI	169	2,608	-	-	163	146	-	-

Abbreviations: CCI, Charlson Comorbidity Index; HIV, Human immunodeficiency virus; SMD, standardized mean difference.

a TriNetX does not provide exact numbers if less than 10 to protect against identification.

**Table 3.** Subtalar Arthrodesis Nonunion Rates After Prior Ankle Arthrodesis Compared With Isolated Subtalar Arthrodesis.

	Ankle-ST (n)	ST only (n)	Ankle-ST Nonunion Rate (%)	ST Nonunion Rate (%)	*P* Value	RR
Total	262	262	30.5	18.7	.002	1.6
Successful	144	144	16.7	16.0	.873	1.04
Failed	121	121	47.9	14.9	<.001	3.0

Abbreviations: RR, risk ratio; ST, subtalar.

**Table 4.** Ankle Arthrodesis Nonunion Rates After Prior Subtalar Arthrodesis Compared With Isolated Ankle Arthrodesis.

	ST-ankle (n)	Ankle only (n)	ST-Ankle Nonunion Rate (%)	Ankle Nonunion Rate (%)	*P* Value	RR
Total	171	171	27.5	19.3	.074	1.4
Successful	109	109	11.1	19.4	.089	0.57
Failed	57	57	56.1	19.3	<.001	2.4

Abbreviations: RR, risk ratio; ST, subtalar.

Of the 171 patients in the ST-Ankle group, 109 patients had a successful primary subtalar fusion, 57 had a failed primary subtalar arthrodesis requiring revision, and 5 had a failed primary subtalar arthrodesis without revision.

In the subanalysis of the successful ST-Ankle cohort, the 109 patients were compared to patients in the Ankle-only cohort. Before matching, the successful ST-Ankle cohort had a higher prevalence of diabetes mellitus (28.4% vs 19.6%, *P* = .021), pulmonary disease (18.3% vs 10.3%, *P* = .006), and renal disease (14.7% vs 7.5%, *P* = .005). After propensity score matching, each group consisted of 109 patients (Supplemental 4). There were no differences in rate of nonunion (11.1% vs 19.4%, *P* = .089) between the successful ST-Ankle and the Ankle-only groups, therefore failing to reject the null hypothesis.

Next, a total of 57 patients in the failed ST-Ankle cohort were identified and compared to the Ankle-only cohort. Before matching, the failed ST-Ankle cohort had a significantly higher prevalence of pulmonary disease (24.6% vs 10.3%, *P* < .001). After propensity score matching, each group consisted of 57 patients (Supplemental 5). The failed ST-Ankle arthrodesis cohort had a significantly higher rate of nonunion (56.1% vs 19.3%, p < 0.001).

Additionally, the failed ST-Ankle group had a 2.4 times greater risk of developing nonunion after subsequent ankle arthrodesis than the successful ST-Ankle group (RR = 2.4, 95% CI = 1.30-4.42).

## Discussion

In this large, propensity-matched cohort, the presence of a prior *failed* ipsilateral arthrodesis, rather than prior fusion itself, was the main driver of increased nonunion risk in a subsequent ankle or subtalar fusion. When the initial arthrodesis healed successfully, nonunion rates for subsequent adjacent fusions were similar to those of isolated procedures. These findings help explain inconsistencies in earlier literature that did not separate failed from successful prior fusions.

### Ankle-ST vs Subtalar-only

Our initial analysis of all comers found significantly higher nonunion rates for patients undergoing subtalar arthrodesis after a prior ankle arthrodesis. This was before we differentiated between patients with failed or successful prior arthrodesis. Once patients with a failed ankle arthrodesis are excluded, outcomes between successful Ankle-ST and ST-Only groups are comparable. In our subanalyses, we found that patients with a history of a failed prior ankle arthrodesis were at a 3-times increased risk of going on to subtalar joint nonunion compared with those patients with a successful ankle fusion. In fact, when patients with a prior failed ankle arthrodesis were excluded, the successful Ankle-ST and ST-Only groups demonstrated comparable nonunion rates. To our knowledge, this is the first time that this finding has been presented in the literature to date.

Prior to propensity score matching, patients in the Ankle-ST cohort exhibited a higher mean BMI and a greater prevalence of comorbidities such as diabetes mellitus, pulmonary disease, renal disease, heart failure, and tobacco use compared with those undergoing isolated subtalar arthrodesis. These findings indicate that patients requiring both ankle and subtalar fusions are often more medically complex, which may reflect more severe underlying pathology or functional impairment. This higher comorbidity burden underscores the clinical relevance of optimizing modifiable risk factors and tailoring perioperative management strategies for patients who undergo multiple hindfoot fusion procedures.

Multiple previous studies have cited significantly higher nonunion rates for subtalar arthrodesis in patients with a preexisting ankle arthrodesis. A retrospective review of 71 patients by Jennison et al^11^ demonstrated a successful union rate of 44.4% in patients with a preexisting ankle arthrodesis and 86.8% in patients without an ankle arthrodesis. On multivariable logistic regression, adjacent ankle arthrodesis was the only significant risk factor for nonunion after subtalar arthrodesis, with an odds ratio of 4.90. Another retrospective review of 151 cases by Zanolli et al^23^ similarly found a significantly higher union rate in patients undergoing subtalar arthrodesis without previous ipsilateral ankle arthrodesis (91.3% vs 61.5%, *P* = .007). In this study, all 13 patients had a successful previous ankle fusion before undergoing subtalar arthrodesis. Most recently, in a retrospective review of 26 patients, Suri et al^18^ demonstrated higher rates of delayed subtalar union (57% vs 33%) and nonunion (50% vs 25%) in patients with previous ankle arthrodesis. Elhassan et al analyzed 133 primary isolated subtalar arthrodeses and found that patients with pre-existing ipsilateral ankle arthrodesis had significantly lower fusion rates compared to those without (52.4% vs 86.9%, *P* = .001). Notably, factors such as age, gender, BMI, smoking status, diabetes, rheumatoid arthritis, and the method of ankle arthrodesis did not significantly influence the outcome, underscoring the potential impact of prior ankle fusion itself on subtalar healing.^8^ These studies did not specify between successful and failed prior ankle arthrodesis. One proposed reason for the higher nonunion rate is disruption of talar blood supply and the presence of avascular subchondral bone, both known risk factors for nonunion after subtalar arthrodesis.^7^ Additionally, some researchers hypothesize that the increased nonunion rates could be due to increased mobility, joint contact forces, and strain in the subtalar joint following ankle arthrodesis.^7,17,19^ However, neither of these biomechanical effects of ankle arthrodesis have been proven in the literature. In fact, a cadaveric study by Wang et al^20^ demonstrated decreased mobility, joint contact forces, and strain in the subtalar joint following ankle arthrodesis.

Our results suggests that although failed ankle arthrodesis increase the risk of subsequent subtalar nonunion, successful ankle fusions do not. These findings help contextualize prior literature and may aid in preoperative risk stratification. Future studies are needed to clarify the biomechanical impact of ankle arthrodesis on subtalar motion and talar blood supply.

### ST-Ankle vs Ankle-only

This retrospective cohort study provides evidence supporting the primary hypothesis that prior successful subtalar arthrodesis followed by subsequent ankle arthrodesis does not inherently increase the risk of nonunion compared to isolated ankle arthrodesis. Following propensity score matching, patients in the successful ST-Ankle cohort demonstrated no statistically significant difference in nonunion rates when compared with those undergoing ankle-only arthrodesis (11.1% vs 19.4%, *P* = .089). These results suggest that when ST fusion heals successfully, it may not adversely affect the outcome of a subsequent ankle arthrodesis and may even be protective in some cases. We initially observed a higher rate of nonunion in patients with a preexisting subtalar arthrodesis undergoing ankle arthrodesis, though not statistically significant. However, when stratified by fusion success, this association was no longer evident, indicating that prior subtalar surgery alone may not independently affect nonunion risk.

Before propensity score matching, patients in the ST-Ankle cohort demonstrated a higher prevalence of comorbidities such as diabetes mellitus, pulmonary disease, and cancer compared with those undergoing isolated ankle arthrodesis. This suggests that patients requiring both subtalar and ankle fusions may represent a clinically more complex and comorbidity-burdened population. The need for multiple procedures in these patients likely reflects more severe underlying pathology or deformity, emphasizing the importance of comprehensive preoperative optimization and careful surgical planning in this high-risk group.

Multiple previous studies have cited significantly higher nonunion rates for ankle arthrodesis in patients with a preexisting subtalar arthrodesis. A retrospective analysis by Woods et al^21^ of 271 patients who underwent an arthroscopic ankle arthrodesis demonstrated that 70% of patients with a preexisting triple arthrodesis went on to nonunion compared to only 5.5% of patients without a preexisting triple arthrodesis (odds radio = 18.3). Of the 22 patients who went on to nonunion, all patients eventually went on to union or opted for no further procedures because of mild or no symptoms. Similarly, a retrospective study by Chalayon et al^3^ found that a prior subtalar fusion was the strongest predictor of nonunion after ankle arthrodesis, with a relative risk of 3.89. However, neither of these studies differentiated patients between subtalar fusion and failed prior subtalar arthrodesis.

The authors of these studies hypothesize that the lower union rates following ankle arthrodesis are caused by the effect of the preexisting subtalar arthrodesis on both the mechanical and biological properties of the ankle joint. The fixed subtalar joint imparts a greater amount of motion to the ankle joint and interferes with the process of bony union after attempted ankle fusion. Additionally, vascular supply to the talus could be partially disrupted during subtalar arthrodesis in some patients, thus impacting the biological propensity for a successful ankle fusion.^3,21^

Prior studies have shown conflicting evidence regarding the biomechanical impact of subtalar arthrodesis on the ankle. Some report decreased ankle range of motion after subtalar fusion, suggesting a more stable, static joint that could favor ankle arthrodesis.^1,24^ However, other studies suggest that although subtalar fusion may reduce overall ankle motion, it alters biomechanics throughout the gait cycle and may lead to compensatory motion at the ankle joint. Hutchinson et al^10^ used cadaveric models to study the effects of hindfoot fusion and found that subtalar arthrodesis reduced total contact force and contact area at the tibiotalar joint during loading in neutral and everted positions. However, it also introduced an external rotation moment at the tibiotalar joint during loading—a change not seen before subtalar fusion. Another study by Palma et al^15^ used robotic gait simulation of the ankle joint and found that decreased ankle motion exists in the sagittal, coronal, and axial planes during early, mid, and late stance of gait following subtalar arthrodesis. However, the authors of this study also found a compensatory increase in internal rotation and inversion of the ankle during the late stance of gait. They hypothesized that fixing the subtalar joint at 5 degrees of valgus may result in decreased hindfoot excursion during push-off and require this compensatory motion of the tibiotalar joint for effective gait.

In this study, we found that preexisting subtalar arthrodesis does not impact union rates after ankle arthrodesis overall. Patients with a prior successful subtalar fusion had similar nonunion rates to the subtalar-only group, whereas those with a failed subtalar arthrodesis had higher rates. A failed subtalar arthrodesis increased the risk of nonunion after ankle arthrodesis by 2.4 times compared with a successful ankle fusion. These findings suggest that while subtalar arthrodesis alone may not affect outcomes, its success or failure is a useful predictor and can aid in risk stratification of patients. Prior biomechanical studies show mixed results regarding the effect of subtalar fusion on the ankle. Future research is needed to better understand how both successful and failed subtalar arthrodesis influence the mechanical and biological conditions that affect ankle fusion, which could guide clinical decision making and patient counseling—particularly when choosing between ankle fusion and total ankle arthroplasty.

These findings provide more context for what has previously been reported in the literature and help in risk-stratifying patients prior to surgery. Future studies are necessary to further understand the biomechanical underpinnings of subtalar fusion and potential consequences of failed and successful subtalar arthrodesis on ankle motion and talar blood supply.

### Limitations

This study has various limitations related to both its internal and external validity. Because *ICD-10* code M96.0 is a billing proxy and not radiographically confirmed, some nonunions were likely undercoded, biasing results toward underestimating the true incidence. Lack of laterality-specific diagnostic codes means some contralateral nonunions could be misclassified as ipsilateral, likely diluting observed associations. Because TriNetX predominantly samples tertiary US academic centers, findings may not generalize to community or safety-net populations. Internal validity is weakened by the study’s multicenter retrospective design, which could result in data collection variability between study sites. Additionally, there is likely some selection bias, because of the retrospective nature of the study and matched analysis, which excluded thousands of patients in both arms of the study. Furthermore, this study required various assumptions that could bias our results. For example, we assumed that the nonunion diagnosis between the index arthrodesis and subsequent arthrodesis was related to the first procedure. An important limitation of this study is the lack of laterality-specific diagnostic codes for nonunion, which prevents definitive attribution of nonunion events to a specific side when patients have undergone bilateral procedures. Although *CPT* codes enabled us to identify the laterality of arthrodesis procedures, this was not possible for subsequent nonunion diagnoses. To mitigate this, we limited our analysis to patients who had both procedures (ankle and subtalar arthrodesis) performed on the same side. However, we could not exclude the possibility that some patients may have undergone contralateral fusions at different time points.

Additionally, we assumed that the earlier fusion had healed by the time of the second procedure, and that nonunion diagnoses occurring between 6 months and 5 years after the second surgery were most likely related to the second, more recent arthrodesis. This assumption introduces some uncertainty and should be considered when interpreting the findings. Also, we did not control for the specific amount of time between the index fusion procedure and subsequent fusion. For example, there could be a difference in the biomechanical and anatomic properties of the subtalar joint 5 years after an ankle arthrodesis compared with 6 months after an ankle arthrodesis. Future studies should be done to elicit the effect of the temporal window between the 2 procedures on subsequent ipsilateral joint fusion rates. The external validity of the study is weakened by the use of a national database, which may yield a higher patient population, but decreases the study’s applicability to any particular real-world population. Although we used a modified CCI that includes a broader range of comorbidities, including peptic ulcer disease, the index has limitations in identifying further conditions that more specifically impact fusion healing. Although relevant comorbidities such as smoking and diabetes are included, the CCI does not capture the severity or control of these conditions (eg, pack-years of smoking or HbA_1c_ levels), which may influence healing outcomes. This lack of clinical granularity represents a limitation of our comorbidity matching protocol. One limitation of our matching analysis is that several variables (BMI, acute myocardial infarction, cerebrovascular accident, and smoking) exceeded the 0.10 SMD threshold. Although SMDs are generally preferred for assessing covariate balance, we also report postmatch *P* values to provide additional transparency; in these cases, the *P* values indicate that differences were not statistically significant, with the exception of acute myocardial infarction and cerebral vascular accident, where TriNetX data suppression for cell counts <10 likely inflated imbalance metrics.

Furthermore, although this study introduces a novel idea, the database analysis does not include in-depth patient-specific information, such as surgical technique, radiographic findings, long-term functional outcomes, and patient-reported outcomes. Therefore, database analysis does not allow the authors to further opine on the reasons for the findings. For example, the absence of radiographic information may bias nonunion rates. Furthermore, we are relying solely on *CPT* codes to elucidate clinical outcomes, so any coding inaccuracies could bias our findings. A key limitation of using administrative databases is the potential for misclassification bias, as *ICD-10* codes are billing-based and do not provide imaging or clinical confirmation. This may result in underrecognition of nonunion events, potentially underestimating the true incidence. Moreover, because TriNetX predominantly samples tertiary US academic centers, results may not generalize to patients treated in community or safety-net hospital settings.

Finally, a limitation of this study is the inability to verify how nonunion was diagnosed—whether by radiographs, CT imaging, clinical symptoms, or intraoperative findings—which may introduce variability in case classification as well as potential for misclassification.

## Conclusion

In this matched cohort study, we found that when the initial arthrodesis was successful, there was no significant difference in nonunion rates for subsequent arthrodesis of an adjacent foot joint compared with isolated procedures. However, failure of prior arthrodesis was associated with an increased risk of nonunion following subsequent adjacent arthrodesis. These findings introduce a novel concept to the current body of literature and may have significant implications on clinical decision making and patient counseling. Our results suggest that a prior successful fusion does not compromise the biological environment for healing in a subsequent adjacent joint arthrodesis. This indicates that such prior procedures should not be considered a contraindication to future procedures. In contrast, patients with a failed prior arthrodesis, even if ultimately revised, demonstrate an elevated risk of nonunion in subsequent arthrodesis. This distinction underscores the need for heightened surgical planning, risk factor optimization, and consideration of adjunctive fusion-enhancing techniques in patients with a history of fusion failure. Future studies should focus on further elucidating the biological and/or biomechanical underpinnings of these processes and clinical outcomes.

## Supplemental Material

sj-docx-2-fai-10.1177_10711007251376296 – Supplemental material for Impact of Prior Ipsilateral Arthrodesis on Subsequent Ankle and Subtalar Fusion Outcomes: A Propensity-Matched Cohort Study Supplemental material, sj-docx-2-fai-10.1177_10711007251376296 for Impact of Prior Ipsilateral Arthrodesis on Subsequent Ankle and Subtalar Fusion Outcomes: A Propensity-Matched Cohort Study by Avani A. Chopra, Kush Mody, Mark Fisher, David Ahn, Gnaneswar Chundi, Abhiram Dawar, Tuckerman Jones, Scott Tucker, Sheldon Lin and Michael Aynardi in Foot & Ankle International

sj-docx-3-fai-10.1177_10711007251376296 – Supplemental material for Impact of Prior Ipsilateral Arthrodesis on Subsequent Ankle and Subtalar Fusion Outcomes: A Propensity-Matched Cohort Study Supplemental material, sj-docx-3-fai-10.1177_10711007251376296 for Impact of Prior Ipsilateral Arthrodesis on Subsequent Ankle and Subtalar Fusion Outcomes: A Propensity-Matched Cohort Study by Avani A. Chopra, Kush Mody, Mark Fisher, David Ahn, Gnaneswar Chundi, Abhiram Dawar, Tuckerman Jones, Scott Tucker, Sheldon Lin and Michael Aynardi in Foot & Ankle International

sj-docx-4-fai-10.1177_10711007251376296 – Supplemental material for Impact of Prior Ipsilateral Arthrodesis on Subsequent Ankle and Subtalar Fusion Outcomes: A Propensity-Matched Cohort Study Supplemental material, sj-docx-4-fai-10.1177_10711007251376296 for Impact of Prior Ipsilateral Arthrodesis on Subsequent Ankle and Subtalar Fusion Outcomes: A Propensity-Matched Cohort Study by Avani A. Chopra, Kush Mody, Mark Fisher, David Ahn, Gnaneswar Chundi, Abhiram Dawar, Tuckerman Jones, Scott Tucker, Sheldon Lin and Michael Aynardi in Foot & Ankle International

sj-docx-5-fai-10.1177_10711007251376296 – Supplemental material for Impact of Prior Ipsilateral Arthrodesis on Subsequent Ankle and Subtalar Fusion Outcomes: A Propensity-Matched Cohort Study Supplemental material, sj-docx-5-fai-10.1177_10711007251376296 for Impact of Prior Ipsilateral Arthrodesis on Subsequent Ankle and Subtalar Fusion Outcomes: A Propensity-Matched Cohort Study by Avani A. Chopra, Kush Mody, Mark Fisher, David Ahn, Gnaneswar Chundi, Abhiram Dawar, Tuckerman Jones, Scott Tucker, Sheldon Lin and Michael Aynardi in Foot & Ankle International

sj-docx-6-fai-10.1177_10711007251376296 – Supplemental material for Impact of Prior Ipsilateral Arthrodesis on Subsequent Ankle and Subtalar Fusion Outcomes: A Propensity-Matched Cohort Study Supplemental material, sj-docx-6-fai-10.1177_10711007251376296 for Impact of Prior Ipsilateral Arthrodesis on Subsequent Ankle and Subtalar Fusion Outcomes: A Propensity-Matched Cohort Study by Avani A. Chopra, Kush Mody, Mark Fisher, David Ahn, Gnaneswar Chundi, Abhiram Dawar, Tuckerman Jones, Scott Tucker, Sheldon Lin and Michael Aynardi in Foot & Ankle International

sj-pdf-1-fai-10.1177_10711007251376296 – Supplemental material for Impact of Prior Ipsilateral Arthrodesis on Subsequent Ankle and Subtalar Fusion Outcomes: A Propensity-Matched Cohort Study Supplemental material, sj-pdf-1-fai-10.1177_10711007251376296 for Impact of Prior Ipsilateral Arthrodesis on Subsequent Ankle and Subtalar Fusion Outcomes: A Propensity-Matched Cohort Study by Avani A. Chopra, Kush Mody, Mark Fisher, David Ahn, Gnaneswar Chundi, Abhiram Dawar, Tuckerman Jones, Scott Tucker, Sheldon Lin and Michael Aynardi in Foot & Ankle International
