# Supplementary material for: Impact of Prior Ipsilateral Arthrodesis on Subsequent Ankle and Subtalar Fusion Outcomes: A Propensity-Matched Cohort Study
Source: Foot Ankle Int. 2025 Nov 5;46(12):1340–50. doi: 10.1177/10711007251376296 (PMC12708960; doi:10.1177/10711007251376296)
Supplement: sj-docx-2-fai-10.1177_10711007251376296 – Supplemental material for Impact of Prior Ipsilateral Arthrodesis on Subsequent Ankle and Subtalar Fusion Outcomes: A Propensity-Matched Cohort Study [file sj-docx-2-fai-10.1177_10711007251376296.docx]

|  |  |  |
| --- | --- | --- |
| Condition | ICD-10 code | Weight |
| Acute myocardial infarction | I21 | 1 |
| Congestive heart failure | I50 | 1 |
| Peripheral vascular disease | I73 | 1 |
| Cerebral vascular accident | I63 | 1 |
| Dementia | F03 | 1 |
| Pulmonary disease | J40-J4A | 1 |
| Connective tissue disorder | M30-M36 | 1 |
| Peptic ulcer | K25 | 1 |
| Liver disease | K70-K77 | 1 |
| Diabetes mellitus | E08-E13 | 1 |
| Hemiplegia | G81.9 | 2 |
| Renal disease | N18 | 2 |
| Cancer | C00-D49 | 2 |
| HIV | B20 | 6 |
|  |  |  |
| Charlson comorbidity index (CCI) equals the sum of the weighted score. Due to limitations of TriNetX database, severity of condition was not included.  HIV: Human immunodeficiency virus, ICD-10: International Classification of Disease 10th Revision | | |

**Supplemental 1:** ICD-10 coding for modified CCI
