# Supplementary material for: Impact of Prior Ipsilateral Arthrodesis on Subsequent Ankle and Subtalar Fusion Outcomes: A Propensity-Matched Cohort Study
Source: Foot Ankle Int. 2025 Nov 5;46(12):1340–50. doi: 10.1177/10711007251376296 (PMC12708960; doi:10.1177/10711007251376296)
Supplement: sj-docx-3-fai-10.1177_10711007251376296 – Supplemental material for Impact of Prior Ipsilateral Arthrodesis on Subsequent Ankle and Subtalar Fusion Outcomes: A Propensity-Matched Cohort Study [file sj-docx-3-fai-10.1177_10711007251376296.docx]

| **Characteristics** | **Before Matching** | | | **After Matching** | | |
| --- | --- | --- | --- | --- | --- | --- |
|  | **Successful  ankle-subtalar** | **Subtalar-only** | **p-value** | **Successful  ankle-subtalar** | **Subtalar-only** | **p-value** |
| Age (years), mean | 54.4 | 51.9 | 0.062 | 54.3 | 55.3 | 0.495 |
| BMI, mean | 34.8 | 32.2 | 0.002 | 34.8 | 34.3 | 0.668 |
| Male, n (%) | 84 (50.9) | 5301 (44.4) | 0.095 | 83 (50.6) | 84 (51.2) | 0.912 |
| Female, n (%) | 77 (46.7) | 6116 (51.2) | 0.243 | 77 (47.0) | 76 (46.3) | 0.912 |
| Acute myocardial infarction, n (%) | 0 (0.0) | 27 (0.2) | 0.541 | 0 (0.0) | 0 (0.0) | - |
| Cancer, n (%) | <10 (6.1)* | 244 (2.0) | <0.001 | <10 (6.1)* | <10 (6.1)* | 1 |
| Cerebral vascular accident, n (%) | 0 (0.0) | 30 (0.3) | 0.519 | 0 (0.0) | 0 (0.0) | - |
| Congestive heart failure, n (%) | <10 (6.1)* | 211 (1.8) | <0.001 | <10 (6.1)* | <10 (6.1)* | 1 |
| Connective tissue disorder, n (%) | <10 (6.1)* | 127 (1.1) | <0.001 | <10 (6.1)* | <10 (6.1)* | 1 |
| Dementia, n (%) | 0 (0.0) | <10 (0.1)* | 0.71 | 0 (0.0) | 0 (0.0) | - |
| Diabetes mellitus, n (%) | 31 (18.8) | 1534 (12.9) | 0.024 | 30 (18.3) | 29 (17.7) | 0.886 |
| Hemiplegia, n (%) | 0 (0.0) | 13 (0.1) | 0.671 | 0 (0.0) | 0 (0.0) | - |
| HIV, n (%) | <10 (6.1)* | 21 (0.2) | <0.001 | <10 (6.1)* | <10 (6.1)* | 1 |
| Liver disease, n (%) | <10 (6.1)* | 110 (0.9) | <0.001 | <10 (6.1)* | <10 (6.1)* | 1 |
| Peptic ulcer, n (%) | 0 (0.0) | 15 (0.1) | 0.649 | 0 (0.0) | 0 (0.0) | - |
| Peripheral vascular disease, n (%) | <10 (6.1)* | 111 (0.9) | <0.001 | <10 (6.1)* | <10 (6.1)* | 1 |
| Pulmonary disease, n (%) | 19 (11.5) | 1192 (10.0) | 0.516 | 19 (11.6) | 21 (12.8) | 0.736 |
| Renal disease, n (%) | 14 (8.5) | 372 (3.1) | <0.001 | 13 (7.9) | 12 (7.3) | 0.835 |
| Tobacco Use, n (%) | 11 (6.7) | 868 (7.3) | 0.766 | 11 (6.7) | <10 (6.1)* | 0.822 |
| Estimated CCI | 198 | 4,751 | - | 195 | 194 | - |
|  |  |  |  |  |  |  |
| HIV: Human immunodeficiency virus, CCI: Charlson Comorbidity Index *TriNetX does not provide exact numbers if less than 10 to protect against identification. | | | | | | |

**Supplemental 2:** Characteristics of patients in successful ankle-subtalar and subtalar-only cohorts before and after matching
