# Supplementary material for: Impact of Prior Ipsilateral Arthrodesis on Subsequent Ankle and Subtalar Fusion Outcomes: A Propensity-Matched Cohort Study
Source: Foot Ankle Int. 2025 Nov 5;46(12):1340–50. doi: 10.1177/10711007251376296 (PMC12708960; doi:10.1177/10711007251376296)
Supplement: sj-docx-4-fai-10.1177_10711007251376296 – Supplemental material for Impact of Prior Ipsilateral Arthrodesis on Subsequent Ankle and Subtalar Fusion Outcomes: A Propensity-Matched Cohort Study [file sj-docx-4-fai-10.1177_10711007251376296.docx]

| **Characteristics** | **Before Matching** | | | **After Matching** | | |
| --- | --- | --- | --- | --- | --- | --- |
|  | **Failed  ankle-subtalar** | **Subtalar-only** | **p-value** | **Failed  ankle-subtalar** | **Subtalar-only** | **p-value** |
| Age (years), mean | 55.2 | 51.9 | 0.044 | 55 | 56 | 0.592 |
| BMI, mean | 34.6 | 32.3 | 0.023 | 34.6 | 32.2 | 0.087 |
| Male, n (%) | 60 (53.6) | 4945 (44.4) | 0.052 | 59 (53.2) | 67 (60.4) | 0.278 |
| Female, n (%) | 46 (41.1) | 5674 (50.9) | 0.038 | 46 (41.4) | 40 (36.0) | 0.408 |
| Acute myocardial infarction, n (%) | 0 (0.0) | 27 (0.2) | 0.602 | 0 (0.0) | 0 (0.0) | - |
| Cancer, n (%) | <10 (8.9)* | 238 (2.1) | <0.001 | <10 (9.0)* | <10 (9.0)* | 1 |
| Cerebral vascular accident, n (%) | 0 (0.0) | 28 (0.3) | 0.595 | 0 (0.0) | 0 (0.0) | - |
| Congestive heart failure, n (%) | <10 (8.9)* | 204 (1.8) | <0.001 | <10 (9.0)* | <10 (9.0)* | 1 |
| Connective tissue disorder, n (%) | 0 (0.0) | 124 (1.1) | 0.261 | 0 (0.0) | 0 (0.0) | - |
| Dementia, n (%) | 0 (0.0) | <10 (0.1)* | 0.751 | 0 (0.0) | 0 (0.0) | - |
| Diabetes mellitus, n (%) | 20 (17.9) | 1459 (13.1) | 0.138 | 20 (18.0) | 16 (14.4) | 0.466 |
| Hemiplegia, n (%) | 0 (0.0) | 12 (0.1) | 0.728 | 0 (0.0) | 0 (0.0) | - |
| HIV, n (%) | 0 (0.0) | 20 (0.2) | 0.654 | 0 (0.0) | 0 (0.0) | - |
| Liver disease, n (%) | <10 (8.9)* | 108 (1.0) | <0.001 | <10 (9.0)* | 0 (0.0) | 0.001 |
| Peptic ulcer, n (%) | <10 (8.9)* | 16 (0.1) | <0.001 | <10 (9.0)* | 0 (0.0) | 0.001 |
| Peripheral vascular disease, n (%) | <10 (8.9)* | 110 (1.0) | <0.001 | <10 (9.0)* | <10 (9.0)* | 1 |
| Pulmonary disease, n (%) | 24 (21.4) | 1123 (10.1) | <0.001 | 23 (20.7) | 27 (24.3) | 0.52 |
| Renal disease, n (%) | 11 (9.8) | 360 (3.2) | <0.001 | <10 (9.0)* | <10 (9.0)* | 1 |
| Tobacco Use, n (%) | 13 (11.6) | 831 (7.5) | 0.097 | 12 (10.8) | 13 (11.7) | 0.832 |
| Estimated CCI | 126 | 4,549 | - | 123 | 103 | - |
|  |  |  |  |  |  |  |
| HIV: Human immunodeficiency virus, CCI: Charlson Comorbidity Index *TriNetX does not provide exact numbers if less than 10 to protect against identification. | | | | | | |

**Supplemental 3:** Characteristics of patients in failed ankle-subtalar and subtalar-only cohorts before and after matching
