# Supplementary material for: Impact of Prior Ipsilateral Arthrodesis on Subsequent Ankle and Subtalar Fusion Outcomes: A Propensity-Matched Cohort Study
Source: Foot Ankle Int. 2025 Nov 5;46(12):1340–50. doi: 10.1177/10711007251376296 (PMC12708960; doi:10.1177/10711007251376296)
Supplement: sj-docx-5-fai-10.1177_10711007251376296 – Supplemental material for Impact of Prior Ipsilateral Arthrodesis on Subsequent Ankle and Subtalar Fusion Outcomes: A Propensity-Matched Cohort Study [file sj-docx-5-fai-10.1177_10711007251376296.docx]

| **Characteristics** | **Before Matching** | | | **After Matching** | | |
| --- | --- | --- | --- | --- | --- | --- |
|  | **Successful Subtalar-ankle** | **Ankle Only** | **p-value** | **Successful Subtalar-ankle** | **Ankle Only** | **p-value** |
| Age (years), mean | 54.6 | 54.5 | 0.943 | 54.8 | 56.2 | 0.421 |
| BMI, mean | 31.8 | 32.2 | 0.688 | 31.8 | 33.3 | 0.312 |
| Male, n (%) | 61 (43.9) | 3457 (54.4) | 0.013 | 61 (44.2) | 56 (40.6) | 0.543 |
| Female, n (%) | 77 (55.4) | 2737 (43.1) | 0.004 | 76 (55.1) | 81 (58.7) | 0.543 |
| Acute myocardial infarction, n (%) | 0 (0.0) | 20 (0.3) | 0.508 | 0 (0.0) | 0 (0.0) | - |
| Cancer, n (%) | <10 (7.2)* | 113 (1.8) | <0.001 | <10 (7.2)* | <10 (7.2)* | 1 |
| Cerebral vascular accident, n (%) | 0 (0.0) | <10 (0.2)* | 0.64 | 0 (0.0) | 0 (0.0) | - |
| Congestive heart failure, n (%) | <10 (7.2)* | 125 (2.0) | <0.001 | <10 (7.2)* | <10 (7.2)* | 1 |
| Connective tissue disorder, n (%) | <10 (7.2)* | 57 (0.9) | <0.001 | <10 (7.2)* | <10 (7.2)* | 1 |
| Dementia, n (%) | 0 (0.0) | <10 (0.2)* | 0.64 | 0 (0.0) | 0 (0.0) | - |
| Diabetes mellitus, n (%) | 31 (22.3) | 864 (13.6) | 0.003 | 31 (22.5) | 32 (23.2) | 0.886 |
| Hemiplegia, n (%) | 0 (0.0) | <10 (0.2)* | 0.64 | 0 (0.0) | 0 (0.0) | - |
| HIV, n (%) | 0 (0.0) | <10 (0.2)* | 0.64 | 0 (0.0) | 0 (0.0) | - |
| Liver disease, n (%) | <10 (7.2)* | 64 (1.0) | <0.001 | <10 (7.2)* | <10 (7.2)* | 1 |
| Peptic ulcer, n (%) | <10 (7.2)* | <10 (0.2)* | <0.001 | <10 (7.2)* | 0 (0.0) | 0.001 |
| Peripheral vascular disease, n (%) | <10 (7.2)* | 55 (0.9) | <0.001 | <10 (7.2)* | 0 (0.0) | 0.001 |
| Pulmonary disease, n (%) | 21 (15.1) | 545 (8.6) | 0.007 | 20 (14.5) | 21 (15.2) | 0.866 |
| Renal disease, n (%) | <10 (7.2)* | 256 (4.0) | 0.063 | <10 (7.2)* | <10 (7.2)* | 1 |
| Tobacco Use, n (%) | <10 (7.2)* | 518 (8.2) | 0.681 | <10 (7.2)* | <10 (7.2)* | 1 |
| Estimated CCI | 142 | 2,578 | - | 141 | 123 | - |
|  |  |  |  |  |  |  |
| HIV: Human immunodeficiency virus, CCI: Charlson Comorbidity Index *TriNetX does not provide exact numbers if less than 10 to protect against identification. | | | | | | |

**Supplemental 4:** Characteristics of patients in successful subtalar-ankle and ankle-only cohorts before and after matching
