# Supplementary material for: Impact of Prior Ipsilateral Arthrodesis on Subsequent Ankle and Subtalar Fusion Outcomes: A Propensity-Matched Cohort Study
Source: Foot Ankle Int. 2025 Nov 5;46(12):1340–50. doi: 10.1177/10711007251376296 (PMC12708960; doi:10.1177/10711007251376296)
Supplement: sj-docx-6-fai-10.1177_10711007251376296 – Supplemental material for Impact of Prior Ipsilateral Arthrodesis on Subsequent Ankle and Subtalar Fusion Outcomes: A Propensity-Matched Cohort Study [file sj-docx-6-fai-10.1177_10711007251376296.docx]

| **Characteristics** | **Before Matching** | | | **After Matching** | | |
| --- | --- | --- | --- | --- | --- | --- |
|  | **Failed Subtalar-ankle** | **Ankle-only** | **p-value** | **Failed Subtalar-ankle** | **Ankle-only** | **p-value** |
| Age (years), mean | 53.2 | 54.5 | 0.537 | 53.5 | 54.4 | 0.676 |
| BMI, mean | 33.5 | 32.2 | 0.316 | 33.2 | 32.3 | 0.596 |
| Male, n (%) | 27 (48.2) | 3394 (54.4) | 0.354 | 27 (49.1) | 33 (60.0) | 0.251 |
| Female, n (%) | 26 (46.4) | 2688 (43.1) | 0.616 | 25 (45.5) | 19 (34.5) | 0.243 |
| Acute myocardial infarction, n (%) | 0 (0.0) | 20 (0.3) | 0.671 | 0 (0.0) | 0 (0.0) | - |
| Cancer, n (%) | <10 (17.9)* | 113 (1.8) | <0.001 | <10 (18.2)* | <10 (18.2)* | 1 |
| Cerebral vascular accident, n (%) | 0 (0.0) | <10 (0.2)* | 0.764 | 0 (0.0) | 0 (0.0) | - |
| Congestive heart failure, n (%) | <10 (17.9)* | 125 (2.0) | <0.001 | <10 (18.2)* | <10 (18.2)* | 1 |
| Connective tissue disorder, n (%) | <10 (17.9)* | 57 (0.9) | <0.001 | <10 (18.2)* | 0 (0.0) | <0.001 |
| Dementia, n (%) | 0 (0.0) | <10 (0.2)* | 0.764 | 0 (0.0) | 0 (0.0) | - |
| Diabetes mellitus, n (%) | 16 (28.6) | 869 (13.9) | 0.002 | 15 (27.3) | 19 (34.5) | 0.409 |
| Hemiplegia, n (%) | 0 (0.0) | <10 (0.2)* | 0.764 | 0 (0.0) | 0 (0.0) | - |
| HIV, n (%) | 0 (0.0) | <10 (0.2)* | 0.764 | 0 (0.0) | 0 (0.0) | - |
| Liver disease, n (%) | <10 (17.9)* | 64 (1.0) | <0.001 | <10 (18.2)* | <10 (18.2)* | 1 |
| Peptic ulcer, n (%) | 0 (0.0) | <10 (0.2)* | 0.764 | 0 (0.0) | 0 (0.0) | - |
| Peripheral vascular disease, n (%) | <10 (17.9)* | 55 (0.9) | <0.001 | <10 (18.2)* | <10 (18.2)* | 1 |
| Pulmonary disease, n (%) | 11 (19.6) | 553 (8.9) | 0.005 | <10 (18.2)* | <10 (18.2)* | 1 |
| Renal disease, n (%) | <10 (17.9)* | 257 (4.1) | <0.001 | <10 (18.2)* | <10 (18.2)* | 1 |
| Tobacco Use, n (%) | <10 (17.9)* | 520 (8.3) | 0.011 | <10 (18.2)* | <10 (18.2)* | 1 |
| Estimated CCI | 107 | 2,593 | - | 105 | 99 | - |
|  |  |  |  |  |  |  |
| HIV: Human immunodeficiency virus, CCI: Charlson Comorbidity Index *TriNetX does not provide exact numbers if less than 10 to protect against identification. | | | | | | |

**Supplemental 5:** Characteristics of patients in failed subtalar-ankle and ankle-only cohorts before and after matching
